# Isolation of Plant Photosystem II Complexes by Fractional Solubilization

**DOI:** 10.3389/fpls.2015.01100

**Published:** 2015-12-10

**Authors:** Patrycja Haniewicz, Davide Floris, Domenica Farci, Joanna Kirkpatrick, Maria C. Loi, Claudia Büchel, Matthias Bochtler, Dario Piano

**Affiliations:** ^1^Laboratory of Structural Biology, Department of Molecular Biology, International Institute of Molecular and Cell BiologyWarsaw, Poland; ^2^Laboratory of Photosynthesis and Photobiology, Department of Life and Environmental Sciences, University of CagliariCagliari, Italy; ^3^Proteomics Core Facility, European Molecular Biology LaboratoryHeidelberg, Germany; ^4^Laboratory of Plant Cell Physiology, Institute of Molecular Biosciences, Goethe-University FrankfurtFrankfurt am Main, Germany; ^5^Department of Bioinformatics, Institute of Biochemistry and BiophysicsWarsaw, Poland

**Keywords:** photosystem II, PSII-LHCII supercomplex, PSII-LHCII megacomplex, thylakoid membranes, *Nicotiana tabacum*, oligomeric state

## Abstract

Photosystem II (PSII) occurs in different forms and supercomplexes in thylakoid membranes. Using a transplastomic strain of *Nicotiana tabacum* histidine tagged on the subunit PsbE, we have previously shown that a mild extraction protocol with β-dodecylmaltoside enriches PSII characteristic of lamellae and grana margins. Here, we characterize residual granal PSII that is not extracted by this first solubilization step. Using affinity purification, we demonstrate that this PSII fraction consists of PSII-LHCII mega- and supercomplexes, PSII dimers, and PSII monomers, which were separated by gel filtration and functionally characterized. Our findings represent an alternative demonstration of different PSII populations in thylakoid membranes, and they make it possible to prepare PSII-LHCII supercomplexes in high yield.

## Introduction

Oxygenic photosynthesis is one of the key processes sustaining the life on our planet by providing the biosphere with oxygen and sugars. Photosystem II (PSII) is a membrane protein complex that plays an essential role in oxygenic photosynthesis. Sunlight drives the splitting of water into oxygen, electrons, and protons (Cardona et al., 2012), but it also causes photodamage, which is minimized by photoprotection in conditions of excess light and repaired by PSII turnover in all conditions. The distribution of PSII in different complexes within the membranes reflects PSII assembly and repair (Pokorska et al., 2009), as well as optimization for efficient usage of light while avoiding or limiting damage (Boekema et al., 1995; Dekker and Boekema, 2005; Takahashi et al., 2009; Watanabe et al., 2009; Pagliano et al., 2011). In lamellae and the marginal grana, PSII is assembled *de novo* or repaired. Exhausted PSII complexes formed in the grana cores migrate to grana margins and lamellae, while being replaced by new and fully functional complexes (Edelman and Mattoo, 2008; Nixon et al., 2010; Puthiyaveetil et al., 2014; Tomizioli et al., 2014). With respect to PSII, lamellae and grana margins may thus be considered as the “nursery”, while the grana cores act as the “photochemical plant” of thylakoids.

Five main forms of PSII are thought to occur *in vivo*: the monomeric (PSIIm or C), the dimeric (PSIId or C_2_), the incomplete form free of the antenna component CP43 (RC-CP47) and finally the two PSII complexes, consisting of several combinations of C_2_ with the trimeric Light Harvesting Complex II (LHCII), which may bind C_2_ strongly (S) or mildly (M) via the so-called minor antenna complexes (CP24, CP26, and CP29). Their assembly leads to higher photosynthetic units called PSII-LHCII supercomplex (PSIIsc or C_2_S_2_) and megacomplex (PSIImc or C_2_S_2_M_2_; Boekema et al., 1999; Eshaghi et al., 1999; de Bianchi et al., 2008; Caffarri et al., 2009). The five PSII types are localized in different regions of the thylakoid membranes (Danielsson et al., 2006). In particular PSIIm and RC-CP47 are typically localized in the lamellae and peripheral part of the grana, being the main constituents of the “nursery” region, whereas PSIId, C_2_S_2_, C_2_S_2_M_2_ are localized in the grana cores, being the main constituents of the functional side of the thylakoid membranes.

We have recently reported the purification of an inactive form of PSIIm that contains the subunit PsbS and appears as one of the main PSII forms associated with the lamellae region of the thylakoid membranes (Haniewicz et al., 2013). In this work, we now describe the fraction that is not solubilized by the mild-extraction protocol. This fraction, that requires harsher extraction conditions, was characterized in order to reveal whether it may contain the grana complexes opening the way for their isolation and characterization. The material solubilized in the second step is characterized by Blue native polyacrylamide gel electrophoresis (BN-PAGE) and mass spectrometry (MS), which together provide information on native molecular mass and subunit composition. After solubilization, granal thylakoids were also subjected to Immobilized-Metal Affinity Chromatography (IMAC) and subsequent Size Exclusion Chromatography (SEC). In the latter step, PSII complexes, supercomplexes, and megacomplexes could be separated, further characterized and compared using several techniques. This procedure allows the chromatography isolation of preparative amounts of highly pure C, C_2_, C_2_S_2_ particles and represents a step further to their structural and functional characterization. The described procedure also provides a direct biochemical probe of PSII organization and distribution in thylakoid membranes.

## Materials and Methods

### Growth of Tobacco Plants

Plant material was obtained from a transplastomic strain of *Nicotiana tabacum* that carries a hexa-histidine tag at the 5′ end of the gene coding for the PsbE subunit (Fey et al., 2008). The plants were grown for 10–12 weeks with a 50% relative humidity at a constant temperature of 25°C and under a light regime of 12 h/day, with a light intensity of 150–200 μmol photons/(s m^2^).

### Thylakoid Preparation

Thylakoid membranes were purified as reported previously by Fey et al. (2008), but in the last centrifugation step they were resuspended in 20 mM MES–NaOH, pH 6.5; 100 mM NaCl; 5 mM MgCl_2_; 10 mM NaHCO_3_; 12.5% (v/v) glycerol.

### Thylakoids Solubilization and PSII Core Complex Purification by Affinity Chromatography

Thylakoid fractions were obtained routinely and with high reproducibility. Lamellar fractions were obtained by using the procedure reported in Haniewicz et al. (2013). Minimal modifications were introduced with the aim to ensure a complete removal of the peripheral grana (and lamellae) from the grana cores. Briefly, thylakoids membranes where solubilized for 30 min at 4°C and the final chlorophyll concentration was kept in a range between 2 and 3 mg/ml (SL) without major changes. After solubilization, the supernatant was separated from the unsolubilized fraction by spinning at 45000 × *g*. The unsolubilized granal fraction was homogenized and subsequently solubilized for 10 min at 4°C at a chlorophyll concentration of 1 mg/ml (SG), as reported in Fey et al. (2008) for the isolation of PSII complexes with LHC polypeptides bound. In both cases solubilization was carried out using 20 mM β-dodecylmaltoside (β-DDM).

Photosystem II samples were prepared routinely using Ni affinity chromatography. PSII was isolated following the procedure reported in Piano et al. (2010) with the only difference that the washing buffer was free of glycerol and betaine (20 mM MES–NaOH, pH 6.5; 100 mM NaCl; 10 mM NaHCO_3_; 15 mM imidazole) and PSII cores were eluted using 40 mM MES–NaOH, pH 6.5; 20 mM NaCl; 5 mM MgCl_2_; 1 mM CaCl_2_; 10 mM NaHCO_3_; 400 mM imidazole. The washing and the elution buffers contained 0.01% instead of 0.03% (w/v) β-DDM.

### Size Exclusion Chromatography

The eluted fractions from Ni-NTA chromatography were pooled and concentrated using Vivaspin 20 ultrafiltration membranes with 100 kDa cutoff until a final volume of 200 μl. The protein sample was loaded on a home-made column of 80 ml bed volume Superose 6 resin (GE Healthcare) with a diameter of 10 mm leading to highly reproducible separations of specific protein complexes. Protein separation and column pre-equilibration were performed in gel filtration buffer (40 mM MES–NaOH, pH 6.5; 20 mM NaCl; 5 mM MgCl_2_; 1 mM CaCl_2_; 10 mM NaHCO_3_; 0.01% (w/v) β-DDM).

### Absorption Spectroscopy, Chlorophyll Determination, and Yield Calculation

The protein content in thylakoids and purified complexes was calculated referring to the Chl *a* and Chl *b* concentrations from three independent measurements. The analysis was done photometrically in 80% (v/v) acetone using a Pharmacia Biotech Ultrospec 4000 spectrophotometer and Chl concentrations were calculated according to Porra et al. (1989). Yield calculation in thylakoids and PSII complexes was expressed in mg or % of chlorophylls. Measurements were performed on samples from 10 independent purifications starting from different thylakoid stocks. For each of these 10 purifications was calculated the amount of thylakoids and the amounts of the specific PSII complexes isolated (C-PsbS, C, C_2_, C_2_S_2_) expressing them in mg or % of Chls with respect to the initial thylakoid amount. Finally, the amounts for each of the 5 classes (thylakoids, C-PsbS, C, C_2_, C_2_S_2_) were averaged respect to the 10 independent purifications (**Table 4**). The values were expressed as means ± standard deviations.

### Polyacrylamide Gel Electrophoresis

Blue native polyacrylamide gel electrophoresis was routinely used as a reference to cross check the correct SG solubilization, the correct SEC profiles and to select the specific pools to be used for further structural and functional tests. According to Schägger and von Jagow (1991), native electrophoresis was performed using 3–12% (w/v) continuous gradient gels. PSII complexes and thylakoid samples at 0.2 mg Chl/ml were mixed with 0.25 volumes of Coomassie Blue Solution (5% (v/v) serva Blue G, 750 mM aminocaproic acid, 35% (w/v) sucrose). The electrophoresis was carried out at 205 V for 5 h for PSII complexes, while for thylakoids it consisted in a run at 60 V for 12 h. In both cases the run was performed at 4°C. After the electrophoretic run, the gels were stained with Coomassie brilliant blue G250.

### Mass Spectrometry

The BN-PAGE gel bands from the SG samples or from the SEC fractions were excised and analyzed. Samples were processed as described in Farci et al. (2014). Protein groups were assigned to bands, and qualitative estimates of protein abundance were based on an “index” obtained by dividing unweighted spectral counts (spectral count) by protein mass (kDa). Proteins were either excluded (index below 0.25), or divided into groups with indices between 0.25 and 0.50 (marked with + in **Table 3**), 0.50 and 0.75 (marked with ++ in **Table 3**) and indices higher than 0.75 (marked with +++ in **Table 3**). Higher indices indicate greater abundance of the protein in the samples.

### Oxygen Evolution

Oxygen evolution rates under light saturation were measured using a Clark-type electrode (Hansatech, England) at 20°C. In the reaction mixture the samples were added to gel filtration buffer enriched with freshly prepared electron acceptors (1 mM 2,6-dichloro-*p*-benzoquinone and 1 mM ferricyanide). The measurements were carried out at a Chls concentration of 100 μg/ml for thylakoids and 50 μg/ml for PSII samples. Activity was tested with three independent measurements on the same preparation and the values were expressed as means ± standard deviations.

### Electron Microscopy

Size Exclusion Chromatography fractions of different PSII complexes from three independent purifications were checked by Transmission Electron Microscopy (TEM). Samples were diluted in gel filtration buffer and applied on glow-discharged carbon coated copper grids (400 mesh) followed by negative staining using filtered 2% uranyl acetate. Electron microscopy was performed in a CM12 electron microscope (Philips, Eindhoven, Netherlands) operated at 80 kV. Images were recorded under low dose conditions (total dose ∼25*e*-/Å^2^) with a ES500W camera (Gatan, Pleasanton, CA, USA) at a magnification of 110 kx.

## Results

### Composition of SL and SG Fractions

In order to characterize PSII species associated to granal thylakoids, we solubilized membranes in two steps. In the first step, we used a previously described mild and long extraction procedure that selectively solubilizes only the peripheral part of the thylakoids (lamellae and grana margins; SL) leading to the chromatography isolation of monomeric PSII that binds the subunit PsbS (see protocol B samples in Table 1 of Haniewicz et al., 2013). The fraction of thylakoids not solubilized during the preparation of SL was pelleted by centrifugation, resuspended and finally subjected to a second solubilization, leading to samples with a PSII content representative for grana cores (SG) that were then subjected to BN-PAGE.

**FIGURE 1:**
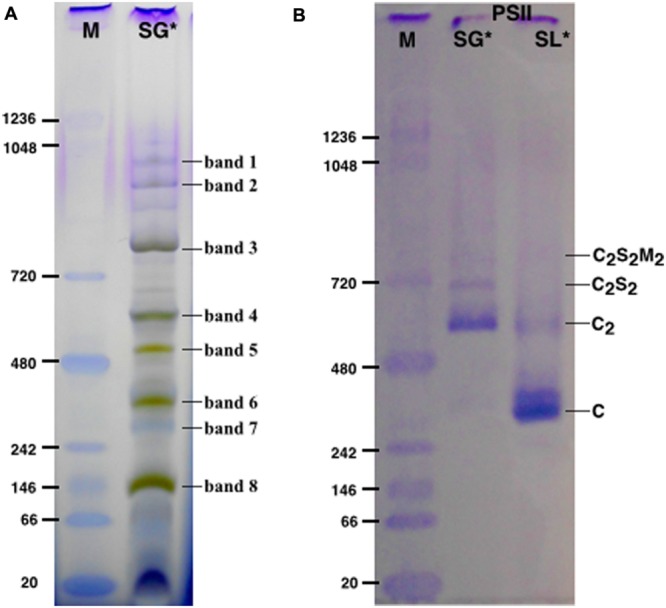
**The solubilized grana cores (SG), when resolved by Blue native polyacrylamide gel electrophoresis (BN-PAGE; **A**) separated into a pattern of bands equivalent to specific thylakoid complexes.** The bands were attributed to a given complex on the basis of their mass spectrometry (MS) analysis (see **Table 1**). The PSII pools purified by NiNTA affinity chromatography are resolved by BN-PAGE **(B)**. The lane SG is the PSII pool purified from the solubilized grana fraction, whereas lane SL represents the pool of PSII purified from solubilized lamellae. In lanes M the molecular marker (M) was loaded. C_2_S_2_M_2_, C_2_S_2_, C_2_ and C are the PSII-LHCII megacomplexes, PSII-LHCII supercomplexes, PSII dimers and PSII monomers, respectively. ^∗^The BN-PAGE used must be considered reliable in the mass range between 66 and 480 kDa according to the molecular marker (M). Above this range, the heaviest bands appear significantly under estimated in weight.

SG samples migrated on BN-PAGE resolving in bands with apparent masses from 1050 to 60 kDa (**Figure 1A**). The content in thylakoid complexes of each band was identified by MS. According to this analysis, PSII was mainly present in the C_2_S_2_M_2_, C_2_S_2_, C_2_ and C forms (**Table 1**, Supplementary Table S1). When incubated on ice for more than 6 h, the SL samples, but not the SG, were characterized by a tendency to precipitate, indicating an insufficient solubilization, which led to difficulties in their characterization by BN-PAGE (data not shown). Taken together, these data suggested large differences in the properties of lamellae and grana cores of the thylakoid membranes.

**Table 1:** Mass spectrometry (MS) analysis performed on the bands of the SG samples resolved by BN-PAGE (see **Figure 1A**).

Band	PSI (LHCI)	PSII (LHCII)	Cyt b_6_f complex	ATP synthase	Complexes
1	PsaB, D, E, F, K CAB4, 7, 40	PsbA, B, C, D, E, O, Q CP29	–	–	PSI and PSIIhigher complexes
2	PsaA, B, D, F, K CAB40	PsbA, B, C, D, Q	–	–	
3	PsaA, B, D, F, L,H,K CAB4,16, 21, 40, lhca	PsbA, B, C, D, E, O CAB7, 36, 40, CP24, 26, 29	–	atpA, B	PSII megacomplex (C_2_S_2_M_2_), PSI-LHCII ATPase
4	PsaA, B, D, E, F, L, CAB4, 25, lhca	PsbA, B, C, D, E, O, S CAB7, 13, 36, 40, CP29, lhcb	–	atpA, B, C, E, F atpα, γ	PSII supercomplex (C_2_S_2_), PSI-LHCII ATPase
5	PsaB CAB16, 21, 40	PsbA, B, C, D, E, O, S CAB7, 13, 36, 50, CP26, 29	–	–	PSII dimer (C_2_) PSI
6	CAB21, 40	PsbA, B, C, D, E, O, Q CAB7, 16, 36, 50, CP26, 29	Cyt. f subunit subunit IV	–	PSII monomer (C), cytb_6_f complex
7	CAB40	PsbA, B, C, D CAB7, 36, CP24, 26	–	–	PSII incomplete monomer
8	CAB21, 40	PsbC, D, O, P, Q, S CAB7, 36, 50, CP24, 26, 29	Cyt. f subunit	atpβ	Free subunits

*For a more detailed analysis see Supplementary Table S1.*

### Oxygen Evolving Activity of SL and SG Fractions

The oxygen evolution capacity of SL and SG samples was tested. The SL samples had a minimal activity of 19 μmol O_2_/mg Chl h, while the SG samples evolved 10-fold more oxygen (186 μmolO_2_/mg Chl h), suggesting the presence of abundant and functional PSII (**Table 2**). These findings are consistent with the accepted view of the lamellae as the assembly and/or repair region of thylakoids in which the PSII particles are mainly inactive, and of the grana in which PSII is in an optimal chemical-physical environment that keeps it very active (Aro et al., 2005).

**Table 2:** Rates of oxygen evolution of solubilized lamellar (SL) thylakoids, solubilized granal (SG) thylakoids and of PSII-LHCII megacomplexes (C2S2M2), PSII-LHCII supercomplexes (C_2_S_2_), PSII dimers (C2), and PSII monomers (C).

Thylakoids^∗^		PSII purified samples^∗^			
SL	SG	PSII monomers (C)	PSII dimers (C_2_)	PSII-LHCII supercomplexes (C_2_S_2_)	PSII-LHCII megacomplexes (C_2_S_2_M_2_)
19 ± 2	186 ± 5	960 ± 5	1360 ± 12	1030 ± 10	not determined

*For each sample, the activity was tested with three independent measurements and the values obtained were expressed in μmol O2/mg Chl h as means ± standard deviations. The C_2_S_2_M_2_ complexes were not characterized because of their low amounts and their C_2_S_2_ impurities. The activity of the C_2_S_2_M_2_ complexes was not determined because of their low amounts and their C_2_S_2_ impurities. ^∗^Values represent means ± standard deviations of three independent measurements.*

### Oligomeric States of PSII Complexes from the SG Fraction

The SG and SL samples were subjected to further PSII separation steps after solubilization. The histidine tag on PsbE of the transplastomic plants made it possible to purify PSII by Ni-NTA (Fey et al., 2008). After purification, the oligomeric profile of the obtained SG-PSII was assessed by BN-PAGE and the composition compared with the already characterized SL-PSII (Haniewicz et al., 2013), showing a significant difference between the oligomeric patterns of the two PSII samples (**Figure 1B**). As clearly shown in **Figure 1B**, mainly C and sometimes incomplete PSII forms such as RC-CP47 can be obtained from SL, while SG is a mixture of C_2_ and C_2_S_2_ with the frequent presence of small C_2_S_2_M_2_ amounts.

### The Different SG-PSII Species can be Separated by Size Exclusion Chromatography

**FIGURE 2:**
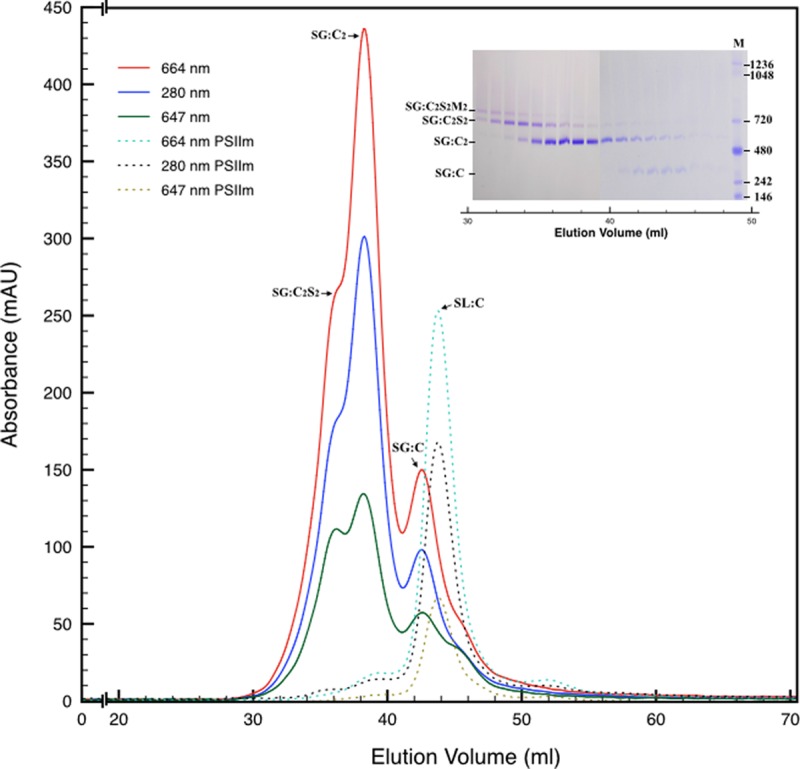
**Size exclusion chromatography of the PSII pool isolated from SG thylakoids by affinity chromatography (solid lines).** All the measurements were recorded at three different wavelengths: 280 nm (proteins), 664 nm (chlorophyll *a*), 647 nm (chlorophyll *b*). In the inset are shown the elution fractions analyzed by BN-PAGE, confirming the partial separation of several oligomeric states of PSII. M is the molecular marker. The mostly monomeric PSII pool isolated from SL thylakoids by affinity chromatography (dotted lines) was used as a mass reference. In the chromatograms and in the inset SG:C_2_S_2_M_2_, SG:C_2_S_2_, SG:C_2_, and SG:C are the PSII-LHCII megacomplexes, PSII-LHCII supercomplexes, PSII dimers, and PSII monomers of grana origin (SG), respectively; SL:C are PSII monomer from lamellae (SL).

**FIGURE 3:**
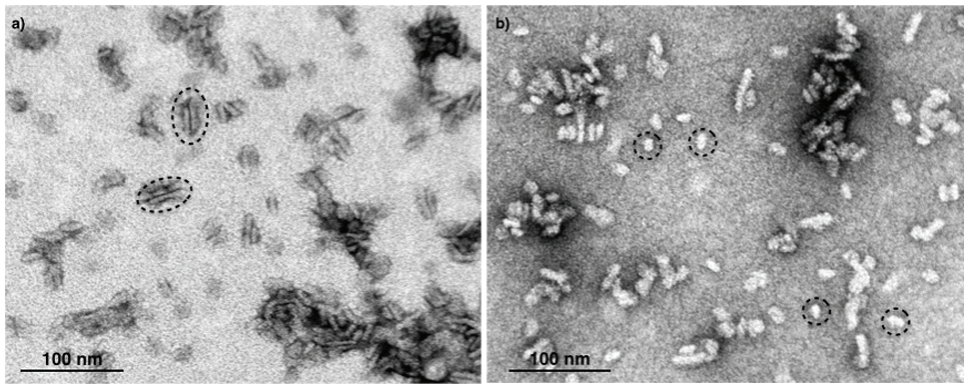
**Electron micrographs of negatively stained C_2_S_2_ complexes **(A)** and C_2_**(B)** separated by size exclusion chromatography.** The suitable fractions in terms of purity were chosen on the basis of their band pattern on BN-PAGE. Dotted ellipses **(A)** and dotted circles **(B)** surround specific examples of C_2_S_2_ complex side views and C_2_ particles, respectively.

Next, we attempted to separate the different PSII components obtained by the Ni-NTA chromatography from the SG-PSII samples by means of SEC. SG-PSII samples resolved into a characteristic and reproducible profile consisting of a shoulder and two peaks (**Figure 2**). In contrast, the same procedure for the SL-PSII samples led to a single peak corresponding to monomeric PSII confirming the BN-PAGE analysis on the NiNTA pool (**Figures 2** and **1B**) and previous results (Haniewicz et al., 2013). The collected fractions from the SEC analysis on the SG-PSII samples were checked by BN-PAGE and thus divided into four pools corresponding to samples enriched in a specific complex primarily identified by its apparent size on BN-PAGE (**Figure 2** inset). The identity of each complex was finally confirmed by MS analysis (**Table 3**, Supplementary Table S2). Furthermore, the C_2_S_2_ and C_2_ complexes were also analyzed by TEM (**Figure 3**), demonstrating the sizes reported for C_2_ and C_2_S_2_ (Aro et al., 2005; Nield and Barber, 2006), the latter most easily discernible from the side views (circled in **Figure 3A**). Unfortunately, C_2_S_2_M_2_ complexes could not be analyzed by TEM, because they were obtained in low amounts and eluted at the beginning of the C_2_S_2_ peak in SEC.

**Table 3:** Mass spectrometry analysis performed on the bands of the SEC fractions resolved by BN-PAGE.

		Name	Accession Number	Mass (kDa)	Qualitative (unweighted subunit presence)				Quantitative^∗^ (weighted subunit presence)			
					l	C_2_	C_2_S_2_	C_2_S_2_M_2_	l	C_2_	C_2_S_2_	C_2_S_2_M_2_
PSII-LHCII (super/megacomplexes)	PSII monomers and dimers	PSB_A (D1)	PSBA_TOBAC	39	+	+	+	+	+++	+++	+++	+++
		PSB_B (CP47)	PSBB_TOBAC	56	+	+	+	+	+++	+++	+++	+++
		PCB_C (CP43)	PSBC_TOBAC	52	+	+	+	+	+++	+++	+++	+++
		PSB_D (D2)	PSBD_TOBAC	40	+	+	+	+	+++	+++	+++	+++
		PSB_E (cytb559)	PSBE_TOBAC	9	+	+	+	+	+++	+++	+++	+
		PSB_H	PSBH_TOBAC	8	+	+	+	+	+++	+++	+++	+++
		PSB_L	PBL_TOBAC	4	–	+	+	–	–	+++	+++	–
		PSB_O (33kDa)	Q84QE8	35	+	+	+	+	–	++	+++	+
		PSB_O (33kDa)	PSBO_TOBAC	35	–	+	+	+	–	++	+++	+
		PSB_R	PSBR_TOBAC	14	–	+	+	+	–	+	+	–
	CAB and LhcII proteins	Lhcb1 (CB24)	CB24_TOBAC	28	+	+	+	+	–	–	+++	+++
		Lhcb1 (CB27)	CB27_TOBAC	28	–	+	+	+	–	–	+++	+++
		Lhcb1 (CB22)	CB22_TOBAC	28	–	–	+	+	–	–	+++	++
		Lhcb1 (CB25)	CB25_TOBAC	28	–	–	+	–	–	–	+++	–
		Lhcb2 (CB23)	CB23_TOBAC (+1)	29	–	+	+	+	–	–	+++	++
		Lhcb3	A0A076L1Y1_TOBAC (+1)	29	–	–	+	+	–	–	–	–
		Lhcb4 (CP29)	Q0PWS7_TOBAC	31	+	+	+	+	–	–	+++	+++
		Lhcb5 (CP26)	Q0PWS5_TOBAC	30	–	+	+	+	–	–	+++	+++
		Lhcb6 (CP24)	Q0PWS6_TOBAC	27	–	+	+	+	–	–	+	+
Plastidial ATP synthase		ATP_A (α-subunit)	ATPA_TOBAC	55	–	+	–	–	–	–	–	–
		ATP_B (β-subunit)	ATPB_TOBAC (+1)	54	–	+	–	–	–	–	–	–
–		37kDa inner membrane polypeptide	Q40501_TOBAC	38	–	+	+	+	–	–	–	–
Vacuolar H^+^-ATPase		subunit B	Q9M5Z8_TOBAC	54	–	–	+	+	–	–	–	–

In the table are shown the protein composition and the relative genes for each of the PSII types separated (**Figure 2** inset). C_2_S_2_M_2_, C_2_S_2_, C_2_, C are the PSII-LHCII megacomplexes, PSII-LHCII supercomplexes, PSII dimers, and PSII monomers, respectively. *Unweighted spectrum count/MASS (kDa) = i; + = 0.25 ≤ i ≤ 0.50; ++ = 0.50 ≤ i ≤ 0.75; +++ = i > 0.75 (for details see Materials and Methods).

### The Isolated PSII Types Evolve Oxygen at Very Different Rates

The different PSII complexes obtained by SEC were tested for their oxygen evolving capacity. The pool of C_2_S_2_M_2_ was excluded from this analysis since, as mentioned above, it was not perfectly separated from the C_2_S_2_. Each one of the others pools, represented by a dominant PSII type, were found to be fully functional and characterized by oxygen evolution rates comparable with other reports in literature (Kihara et al., 2014; **Table 2**). As oxygen evolution per Chl is reported, and because C_2_S_2_ binds about three times more Chls per reaction center than cores (around 210/reaction center as opposed to around 70; Ferreira et al., 2004; Liu et al., 2004; Loll et al., 2005; Barros et al., 2009), the C_2_S_2_ complexes are characterized by the highest activity.

### Mass Spectrometry Analysis on the Four PSII Types Showed Significant Differences in Subunit Composition

The BN-PAGE bands obtained from the SEC fractions (**Figure 2** inset) were excised and directly analyzed by MS to characterize subunit content (**Table 3**, Supplementary Table S2). The main core subunits D1, D2, CP43, CP47, PsbE, PsbH, PsbL, PsbO, and PsbR were found in this analysis. The subunit PsbL, an important dimerization factor (Suorsa et al., 2004), was absent from the C and C_2_S_2_M_2_ types. PsbR was identified only in the C_2_ and C_2_S_2_ types, while PsbF was not found. The partial or complete absence of these three small peptides is most likely due to their small masses, which limit their successful MS identification (Shi et al., 2012). The PsbO subunit was found in both isoforms. The C_2_ and C forms appeared to contain lower amounts of this protein, even though they showed a fair water splitting capacity (**Table 2**). PsbP and PsbQ, the other main water splitting subunits, were not identified in the analyzed complexes. Specific subunits were characteristic for a given oligomeric state. As expected, it was found that the subunits CP24, CP26, and CP29 were present just in traces or absent in the C and C_2_, while they were found to be characteristic for C_2_S_2_ and C_2_S_2_M_2_. Also the proteins Lhcb1, Lhcb2 and Lhcb3 were found associated with the C_2_S_2_ and C_2_S_2_M_2_ complexes. In particular Lhcb3 was found to be present only in traces and Lhcb1 was present in four different isoforms. One of these, CB25, was specific for the C_2_S_2_ form.

## Discussion

### The Oxygen Evolving Properties Reflect the PSII Cycle

The distribution of PSII in thylakoid membranes reflects the PSII cycle (Tomizioli et al., 2014). Grana cores sustain water splitting at the highest rates. Lamellae and grana margins are mainly required to recycle and replace PSII, which at these stages is still exposed to light, but requires protection. In agreement with this general picture, PSII isolated from SL samples is inactive, monomeric and retains PsbS. The presence of PsbS and low PSII activity in SL samples fit with the low activity of lamellar PSII reported earlier and with the role of PsbS in non-photochemical quenching (Allen, 2002; Munekage et al., 2002; Gerotto et al., 2015). PsbS may keep PSII in an inactive and protected state for assembly or repair. PSII from the SG samples is a mix of four different types that are very active in oxygen evolution, also in agreement with the current model for the PSII cycle.

### PsbP and PsbQ

All active PSII complexes from grana lacked the PsbP and PsbQ subunits, which were found earlier in the SL fractions (Haniewicz et al., 2013). As PsbP and PsbQ are thought to enhance the PSII activity and the Oxygen Evolving Complex (OEC) stability (Bricker and Frankel, 2011), this finding is surprising, but it is not unprecedented. PsbP and PsbQ were also absent after a very mild membrane solubilization in a study on D1 subunit processing (Figure 5B from Che et al., 2013). However, the two subunits were present in several earlier PSII preparations, at least judging from SDS-PAGE gel migration patterns (Boekema et al., 2000; Nield et al., 2000; Caffarri et al., 2009). Mummadisetti et al. (2014) also detected PsbP and PsbQ by Western blotting, but, unfortunately, they did not determine the oligomeric PSII state by BN-PAGE and/or SEC. Boekema et al. (1998) detected PsbP in PSII-LHCII supercomplexes, but other studies did not corroborate this finding (Caffarri et al., 2009; Barera et al., 2012). Thus, PsbP and PsbQ may only be present in some assembly stages of PSII complexes or, more likely, in the very active forms of PSII they may be loosely bound due to their high sensitivity to damage.

### Lhcb3 and CP24

Low levels of Lhcb3 and CP24 were detected for both C_2_S_2_M_2_ and C_2_S_2_ complexes. The latter has not been reported to contain these two subunits. However, special hemi-types, intermediate between the C_2_S_2_ and the C_2_S_2_M_2_ types, which retain both extra subunits, have been described and could fit well with the co-presence in our C_2_S_2_ samples. In Caffarri et al. (2009) is reported a so-called C_2_SM complex containing only one S trimer and one M trimer that are bridged through CP29, CP24, and CP26 to the dimeric core. This complex has almost the same mass (1040 kDa) as the C_2_S_2_ complex (1100 kDa), and therefore co-migrates in BN-PAGE (and also co-sediments in a sucrose gradient; Caffarri et al., 2009). Another hemi-type, called C_2_S_2_^+^, is reported to have similar characteristics (Pietrzykowska et al., 2014). It is likely that these incomplete forms are assembly intermediates on the way to C_2_S_2_M_2_, or result from its disintegration, either in membranes or during solubilization or purification.

### Isolation of Supercomplexes by Coupling Differential Solubilization and Chromatography

The organization of the thylakoids and in particular the distribution of pigment–protein complexes within are very well characterized (Danielsson et al., 2006). This and other works all demonstrate substantial heterogeneity of the PSII species in the thylakoid membranes (Boekema et al., 1995; Dekker and Boekema, 2005; Danielsson et al., 2006; Takahashi et al., 2009; Watanabe et al., 2009; Pagliano et al., 2011). Here, we present a differential solubilization of the thylakoid membranes, which leads to the isolation of samples with different oligomeric patterns of PSII and other thylakoid complexes (**Figure 1A**). Conceptually, our method to access to the grana cores fraction, in which is found highly active PSII, is similar to the method described by Berthold et al. (1981), the so-called BBY preparation, which also removes lamellae and peripheral granal regions. In contrast to the BBY method, the new method has the advantage to avoid the breakdown of granal PSII core complexes (C and RC-CP47 are found at most in small quantities).

## Conclusion

It has to be remarked that the mild nature of the solubilization protocol permits a full separation between PSII monomers and dimers, and makes it possible to prepare C_2_S_2_ in larger quantities without density gradients on sucrose or Percoll (Ghanotakis et al., 1987; Caffarri et al., 2004), (**Table 4**).

**Table 4:** Purification yields for the isolated PSII types.

	Thylakoid membranes	Purification				
		Mild solubilization (SL)	Harsh solubilization (SG)			
		C-PsbS	C	C_2_	C_2_S_2_	C_2_S_2_M_2_
Mass (mg)	34.97 ± 3.8	0.95 ± 0.11	0.19 ± 0.01	9.55 ± 0.81	1.91 ± 0.18	Not determined
Yield (%)	100	2.73 ± 0.31	0.55 ± 0.04	27.30 ± 2.33	5.45 ± 0.51	Not determined

*C_2_S_2_M_2_ yield was not determined because of their low amounts and their C_2_S_2_ impurities. ^∗^Values are expressed in mg or % of chlorophylls and represent means ± standard deviations of 10 independent purifications (see details in Materials and Methods).*

## Author Contributions

DP conceived the study, participated in its design and coordination, carried out the membranes preparation, participated in the biochemical studies, and drafted the manuscript. PH participated in the design of the study, participated the biochemical studies, and participated in the membranes preparation. D. Floris carried out the membranes preparation, participated in the biochemical studies. D. Farci carried out the membranes preparation, participated in the biochemical studies. JK carried out the MS analysis. ML participated in the preparation of membranes. CB contributed to the electron microscopy analysis and helped to draft the manuscript. MB participated in the design of the study and helped to draft the manuscript.

## Conflict of Interest Statement

The authors declare that the research was conducted in the absence of any commercial or financial relationships that could be construed as a potential conflict of interest.
