# Supplementary material for: Isolation of Plant Photosystem II Complexes by Fractional Solubilization
Source: Front Plant Sci. 2015 Dec 10;6:1100. doi: 10.3389/fpls.2015.01100 (PMC4674563; doi:10.3389/fpls.2015.01100)
Supplement: Supplementary file 1 [file Table_1.PDF]

		NAME	Accession Number	Mass (kDa)	BN-PAGE BANDs from SG samples qualitative (unweighted subunit presence)							
					1	2	3	4	5	6	7	8
PSI-LHCI complexes	PSI	PSA_A	P06405 (PSAA_TOBAC)	83	+	+	+	+	-	-	-	-
		PSA_B	P06407 (PSAB_TOBAC)	82	+	+	+	+	+	-	-	-
		PSA_D	gi|131167	22	+	+	+	+	-	-	-	-
		PSA_E	gi|1217601 (+1)	16	+	-	-	-	-	-	-	-
		PSA_F	Q9T2G2_TOBAC	2	+	+	+	+	-	-	-	-
		PSA_H	Q84QE6_TOBAC	17	-	-	+	-	-	-	-	-
		PSA_K	Q84QE6_TOBAC	13	+	+	+	+	-	-	-	-
		PSA_L	gi|29468504	23	-	-	+	+	-	-	-	-
	LHCI	Lhca	Q40512_TOBAC	26	-	-	-	+	-	-	-	-
PSII-LHCII (super/megacomplexes)	PSII monomers and dimers	PSB_A (D1)	PSBA_TOBAC	39	+	+	+	+	+	+	+	-
		PSB_B (CP47)	PSBB_TOBAC	56	+	+	+	+	+	+	+	-
		PCB_C (CP43)	PSBC_TOBAC	52	+	+	+	+	+	+	+	+
		PSB_D (D2)	PSBD_TOBAC	40	+	+	+	+	+	+	+	+
		PSB_E (cytb$_{559}$)	PSBE_TOBAC	9	+	-	+	+	+	+	-	-
		PSB_H	PSBH_TOBAC	8	-	-	-	+	-	-	-	-
		PSB_O (33kDa)	Q84QE8	35	+	-	+	+	+	+	-	+
		PSB_P	PSBP2_TOBAC	29	-	-	-	-	-	-	-	+
		PSB_Q	Q5EFR4_TOBAC	24	+	+	-	-	-	+	-	+
		PSB_S	Q9SMB4	29	-	-	-	+	+	-	-	+
	CAB and LHCII	Lhcb (CAB4)	Q40481_TOBAC	22	+	-	+	+	-	-	-	-
		Lhcb1 (CB24)	CB24_TOBAC	28	+	+	+	+	+	+	+	+
		Lhcb1 (CB27)	CB27_TOBAC	28	+	-	+	+	+	+	+	+
		Lhcb1 (CB22)	CB22_TOBAC	28	-	-	+	+	+	+	-	+
		Lhcb1 (CB25)	CB25_TOBAC	28	-	-	-	+	+	+	-	+
		Lhcb1 (CB21)	CB21_TOBAC	28	-	-	+	+	+	+	-	+
		Lhcb2 (CB23)	CB23_TOBAC (+1)	29	-	-	+	+	+	+	+	+
		Lhcb3	A0A076L1Y1_TOBAC (+1)	29	-	-	-	+	+	-	-	+
		Lhcb4 (CP29)	Q0PWS7_TOBAC	31	+	-	+	+	+	+	+	+
		Lhcb5 (CP26)	Q0PWS5_TOBAC	30	-	-	+	-	-	+	+	+
		Lhcb6 (CP24)	Q0PWS6_TOBAC	27	-	-	+	-	-	-	+	+
Plastidial ATP synthase		ATP_A (α-subunit)	ATPA_TOBAC	55	-	-	+	+	-	-	-	-
		ATP_B (β-subunit)	ATPB_TOBAC (+1)	54	-	-	+	+	-	-	-	+
		ATP_C (γ-chain)	ATPG_TOBAC	41	-	-	-	+	-	-	-	-
		ATP_E (ε-chain)	ATPE_TOBAC	15	-	-	-	+	-	-	-	-
		ATP_F (β-subunit)	ATPF_TOBAC	21	-	-	-	+	-	-	-	-
Cytochrome b_6f complex		Cyt f subunit	gi|11465970	35	-	-	-	-	-	+	-	+
		Apocyt f	P06449 (CYF_TOBAC)	35	-	-	-	-	-	-	-	+
		subunit IV	P06249 (PETD_TOBAC)	17	-	-	-	-	-	+	-	-

Sup. Table 1: MS analysis of the SG fraction on the bands resolved by BN-PAGE. For each band are shown the protein composition and the relative genes.
