# Supplementary material for: Isolation of Plant Photosystem II Complexes by Fractional Solubilization
Source: Front Plant Sci. 2015 Dec 10;6:1100. doi: 10.3389/fpls.2015.01100 (PMC4674563; doi:10.3389/fpls.2015.01100)
Supplement: Supplementary file 2 [file Table_2.PDF]

		NAME	Accession Number	Mass (kDa)	Unweighted spectrum counts			
					C	C_2	C_2S_2	$C_2S_2M_2$
PSII-LHCII (super/megacomplexes)	PSII monomers and dimers	**PSB_A (D1)**	PSBA_TOBAC	39	64	164	100	61
		**PSB_B (CP47)**	PSBB_TOBAC	56	91	214	150	70
		**PCB_C (CP43)**	PSBC_TOBAC	52	69	169	147	94
		**PSB_D (D2)**	PSBD_TOBAC	40	42	142	100	62
		**PSB_E (cytb559)**	PSBE_TOBAC	9	9	19	11	3
		**PSB_H**	PSBH_TOBAC	8	8	23	15	9
		**PSB_L**	PBL_TOBAC	4	-	12	7	-
		**PSB_O (33kDa)**	Q84QE8	35	3	21	31	17
		**PSB_O (33kDa)**	PSBO_TOBAC	35	-	19	33	17
		**PSB_R**	PSBR_TOBAC	14	-	5	5	2
	CAB and LhcII proteins	**Lhcb1 (CB24)**	CB24_TOBAC	28	2	3	36	25
		**Lhcb1 (CB27)**	CB27_TOBAC	28	-	3	34	24
		**Lhcb1 (CB22)**	CB22_TOBAC	28	-	-	26	20
		**Lhcb1 (CB25)**	CB25_TOBAC	28	-	-	23	-
		**Lhcb2 (CB23)**	CB23_TOBAC (+1)	29	-	4	28	19
		**Lhcb3**	A0A076L1Y1_TOBAC (+1)	29	-	-	3	3
		**Lhcb4 (CP29)**	Q0PWS7_TOBAC	31	3	6	55	43
		**Lhcb5 (CP26)**	Q0PWS5_TOBAC	30	-	3	34	24
		**Lhcb6 (CP24)**	Q0PWS6_TOBAC	27	-	5	8	12
**Plastidial ATP synthase**		**ATP_A (-subunit)**	ATPA_TOBAC	55	-	5	-	-
		**ATP_B (-subunit)**	ATPB_TOBAC (+1)	54	-	6	-	-
**-**		**37kDa inner membrane polypeptide**	Q40501_TOBAC	38	-	3	3	2
**Vacuolar H^+-ATPase**		**subunit B**	Q9M5Z8_TOBAC	54	-	-	6	6

Supl. Table 2: MS analysis of the bands resolved by BN-PAGE on the SEC fractions. For each complex, the identified subunits are expressed in terms of unweighted spectrum counts as extracted from scaffold. In the table are shown the protein composition and the relative genes for each of the PSII types separated (fig. 2 inset). $C_2S_2M_2$, C_2S_2, C_2, C are the PSII-LHCII megacomplexes, PSII-LHCII supercomplexes, PSII dimers, and PSII monomers, respectively.
